# Virulence Studies of Different Sequence Types and Geographical Origins of *Streptococcus suis* Serotype 2 in a Mouse Model of Infection

**DOI:** 10.3390/pathogens5030048

**Published:** 2016-07-11

**Authors:** Jean-Philippe Auger, Nahuel Fittipaldi, Marie-Odile Benoit-Biancamano, Mariela Segura, Marcelo Gottschalk

**Affiliations:** 1 Swine and Poultry Infectious Diseases Research Center (CRIPA), Department of Pathology and Microbiology, Faculty of Veterinary Medicine, University of Montreal, 3200 Sicotte St., Saint-Hyacinthe, QC J2S 2M2, Canada; jean-philippe.auger.1@umontreal.ca (J.-P.A.); marie-odile.benoit-biancamano@umontreal.ca (M.-O.B.-B.); mariela.segura@umontreal.ca (M.S.); 2 Public Health Ontario and Department of Laboratory Medicine and Pathobiology, Faculty of Medicine, University of Toronto, 661 University Avenue, Toronto, ON M5G 1M1, Canada; nahuel.fittipaldi@oahpp.ca

**Keywords:** *Streptococcus suis* serotype 2, virulence, sequence type, mouse model, inflammation, phagocytosis, blood bactericidal assay

## Abstract

Multilocus sequence typing previously identified three predominant sequence types (STs) of *Streptococcus suis* serotype 2: ST1 strains predominate in Eurasia while North American (NA) strains are generally ST25 and ST28. However, ST25/ST28 and ST1 strains have also been isolated in Asia and NA, respectively. Using a well-standardized mouse model of infection, the virulence of strains belonging to different STs and different geographical origins was evaluated. Results demonstrated that although a certain tendency may be observed, *S. suis* serotype 2 virulence is difficult to predict based on ST and geographical origin alone; strains belonging to the same ST presented important differences of virulence and did not always correlate with origin. The only exception appears to be NA ST28 strains, which were generally less virulent in both systemic and central nervous system (CNS) infection models. Persistent and high levels of bacteremia accompanied by elevated CNS inflammation are required to cause meningitis. Although widely used, in vitro tests such as phagocytosis and killing assays require further standardization in order to be used as predictive tests for evaluating virulence of strains. The use of strains other than archetypal strains has increased our knowledge and understanding of the *S. suis* serotype 2 population dynamics.

## 1. Introduction

*Streptococcus suis* is an important porcine bacterial pathogen and emerging zoonotic agent responsible for sepsis/septic shock and meningitis during systemic and central nervous system (CNS) infections, respectively [1]. Other infections have also been reported in both pigs and humans [2]. Of the different described serotypes based on the presence of the capsular polysaccharide or its respective genes*,* serotype 2 is regarded as not only the most widespread worldwide, but also the most virulent, responsible for the majority of both *S. suis* porcine and human cases of infection [2,3]. However, the global distribution of this serotype is unequal, with cases due to this serotype being much less common in North America (NA) than in Europe and Asia (Eurasia; EA) [2]. Consequently, it has been suggested that virulence amongst *S. suis* serotype 2 strains may vary according to the geographical origin and virulence factors present, subjects that remain widely debated [1,4].

Over the years, different approaches have been used to characterize *S. suis* serotype 2 strains, including the presence or absence of different putative virulence factors or markers [5,6,7]. Alongside, various DNA typing techniques, including random amplification of polymorphic DNA, amplified fragment length polymorphism, restriction fragment length polymorphism, comparative genome hybridization, and pulsed field gel electrophoresis have also been used [8,9,10,11,12]. Of the DNA typing techniques available, multilocus sequence typing (MLST) has emerged as one of the leading methods used for *S. suis* classification, being a reliable, generic, and reproducible technique [13,14,15]. More recently, a number of studies have used MLST as an approach to determining virulence profiles of *S. suis* serotype 2 strains from different sequence types (STs). The clonal ST7 strain responsible for two human outbreaks in China was demonstrated to be much more virulent than archetypal European ST1 strains using mouse models of infection [16,17,18]. Meanwhile, archetypal NA ST25 and ST28 strains were shown to be less virulent than the abovementioned ST1 and ST7 strains [16,19]. Nevertheless, ST25 strains presented higher virulence than their ST28 counterparts [19]. Yet, the actual *S. suis* serotype 2 situation remains misunderstood: around 5% of NA strains (including a strain isolated from a human case) are ST1 [20], a ST typically recovered in EA [2], while strains recovered from certain human cases in Thailand and Japan have been characterized as ST25 or ST28 (commonly isolated in NA), respectively [21,22]. Interestingly, and only adding to this complexity, whole genome sequencing recently revealed that ST25 and ST28 strains do not constitute a highly homogenous group, as was previously believed, since NA and Asian strains clustered separately within each ST [23,24]. Consequently, it remains unclear whether strains of the same ST have similar virulence potentials.

Experimental infections with the natural host, the pig, are possible, though difficult to achieve with a high number of strains [4]. In addition, almost all animals are carriers of *S. suis* in their tonsils, which may influence their response to the infection [4,25]. To overcome this variation, well-established inbred mouse models of *S. suis* infection have been developed, as exemplified by C57BL/6 mice, which reproduce similar clinical signs to those observed during the systemic (septic shock) and CNS (meningitis) infections in the natural host [16,26,27,28]. So far, no study has compared strains belonging to the same ST but from different origins in detail. Therefore, the aim of this study was to evaluate and compare the virulence potential of strains belonging to three important STs (ST1, ST25, and ST28) from different geographical (Americas and EA) and host (porcine and human) origins during both the systemic and CNS infections using the well-established C57BL/6 mouse model of infection.

## 2. Results

### 2.1. Lack of Correlation between In Vitro Resistance to Phagocytosis and to Bacterial Killing by Different S. suis ST Strains

In order to determine the capacity of different *S. suis* serotype 2 strains to resist phagocytosis by murine macrophages, bacteria were incubated with cells and 10% mouse serum for 1 h [29]. As presented in Table 1, the NA and EA ST28 strains were significantly more resistant to phagocytosis than both the ST1 (*p* < 0.05) and ST25 (*p* < 0.001) strains. Differences were not observed in phagocytosis rates between ST1 and ST25 strains nor between NA and EA ST28 strains. As expected, a significant difference was also observed between P1/7 and its non-encapsulated mutant strain, Δ*cpsF*, used as a positive control (*p* < 0.001).

To better simulate the action of the different host phagocytic cells and blood components that bacteria must confront, the bactericidal effect of blood on the different *S. suis* serotype 2 strains was evaluated. As shown in Table 1, and in contradiction to what was observed with phagocytosis, the ST1 strains were completely resistant to bacterial killing by blood leukocytes. This resistance was significantly different (*p* < 0.001) from that of all other STs. Meanwhile, with one exception in each group, the ST25 and EA ST28 strains presented an intermediate level of resistance to the bactericidal effect of blood. On the other hand, the NA ST28 strains were the least resistant to the bactericidal effect, with 30% of bacteria killed on average. In fact, these strains were the only ones to actually be killed in blood, since the number of bacteria recovered in the presence of leukocytes after incubation was lower than that present at the beginning of the incubation time (data not shown). As expected, the high percentage of bacterial killing (64%) of the non-encapsulated mutant strain, Δ*cpsF*, significantly differed from its wild-type strain, P1/7 (*p* < 0.001).

### 2.2. Systemic Infection: An Important Variation of Virulence Among Strains Belonging to the Same STs

**Figure 1:**
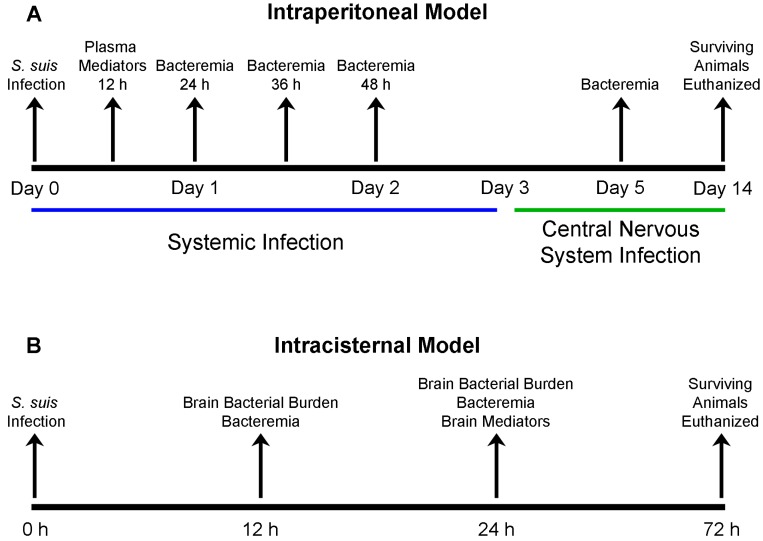
Timeline summary of the intraperitoneal and intracisternal mouse models of infection used throughout this study. C57BL/6 mice were infected with the different *S. suis* serotype 2 strains using the intraperitoneal route of infection (**A**) to evaluate the systemic and subsequent central nervous system infection; or the transcutaneal intracisternal route of infection (**B**) to directly evaluate the central nervous system infection.

A timeline summary of the infections carried out in this study was included (Figure 1). First, the survival of mice intraperitoneally inoculated with the different *S. suis* serotype 2 strains was evaluated until 72 h post-infection (p.i.), during which mortality is the result of sepsis and/or septic shock (Table 1). Interestingly, the in vitro assays used could not (phagocytosis) or only partially (killing) predicted virulence. When taken together, EA ST28, ST1, ST25, and NA ST28 strains caused 50%, 35%, 18%, and 2% of mortality on average, respectively. Indeed, mice infected with the NA ST28 strains presented the lowest mortality, which was significantly different from that of the ST1-, ST25-, and EA ST28-infected mice (*p* < 0.05). In addition, the mortality of ST25-infected mice was significantly lower than that of both ST1- and EA ST28-infected mice (*p* < 0.05), between which there was no difference. No significant difference in mortality recorded at 72 h p.i. was observed between strains of porcine or human origin. However, a significant difference was observed when strains from different geographical origins were compared, Americas versus EA, with 15% and 33% mortality (*p* < 0.001), respectively. Regardless of statistical differences between STs, important variations amongst strains of the same ST were observed. For example, ST1 strains presented mortality rates that ranged between 0% and 80%, ST25 strains between 0% and 47%, and ST28 strains between 0% and 73%.

### 2.3. Bacteremia during the Acute Phase of Systemic Infection: Blood Bacterial Burden is Associated with Mortality Recorded at 72 h p.i. for Most Strains

**Figure 2:**
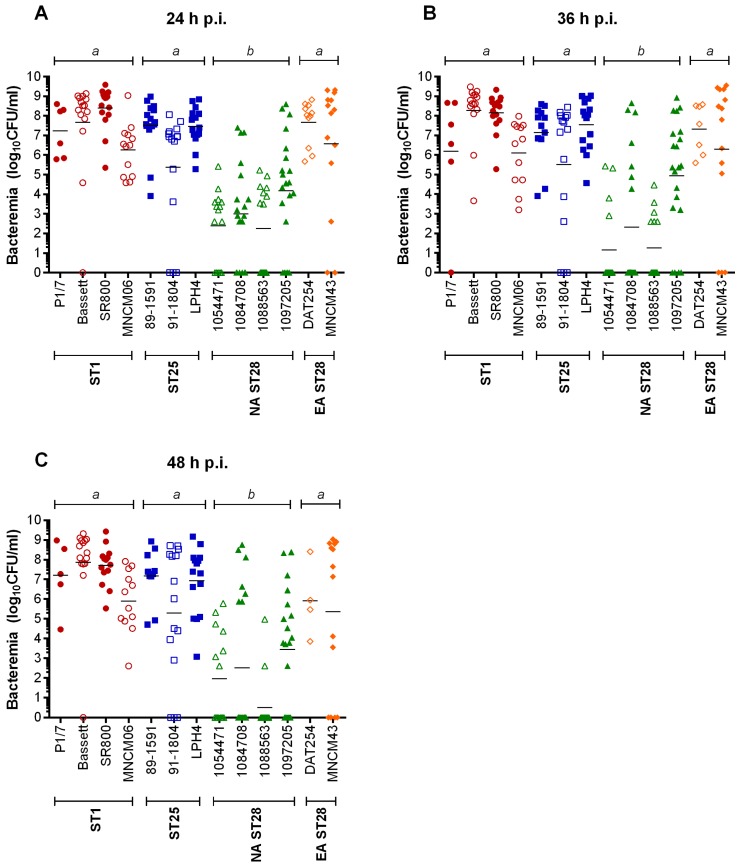
Blood bacterial burden is lower in North American ST28-infected mice but similar in ST1, ST25 or Eurasian ST28-infected mice during the systemic infection. C57BL/6 mice were inoculated by intraperitoneal injection with 5 × 10^7^ CFU and blood bacterial titers evaluated 24 h (**A**); 36 h (**B**) and 48 h (**C**) post-infection (p.i.). Data of individual mice are presented as log_10_ CFU/mL with the geometric mean. Significance between groups is indicated by different letters (*p* < 0.001). Only strains for which five or more mice survived at the indicated time point are presented.

Mortality during the systemic infection caused by *S. suis* may be the consequence of high blood bacterial titers. Consequently, the blood bacterial burden of infected mice was measured at 24 h, 36 h, and 48 h p.i. (Figure 1). At all time points, significant differences were observed between titers of ST1-, ST25-, or EA ST28-infected mice when compared to those infected with NA ST28 strains (*p* < 0.001) (Figure 2). Indeed, it was possible to associate bacteremia with mortality 72 h p.i. for most strains. However, this was not universal, since blood bacterial titers were intermediate or high for Bassett, SR800, and LPH4 strains, while the mortality caused by these strains was low.

### 2.4. Inflammation during the Acute Phase of Systemic Infection: Plasma Pro-Inflammatory Mediator Levels Are Associated with Mortality Recorded at 72 h p.i. for Most Strains

**Figure 3:**
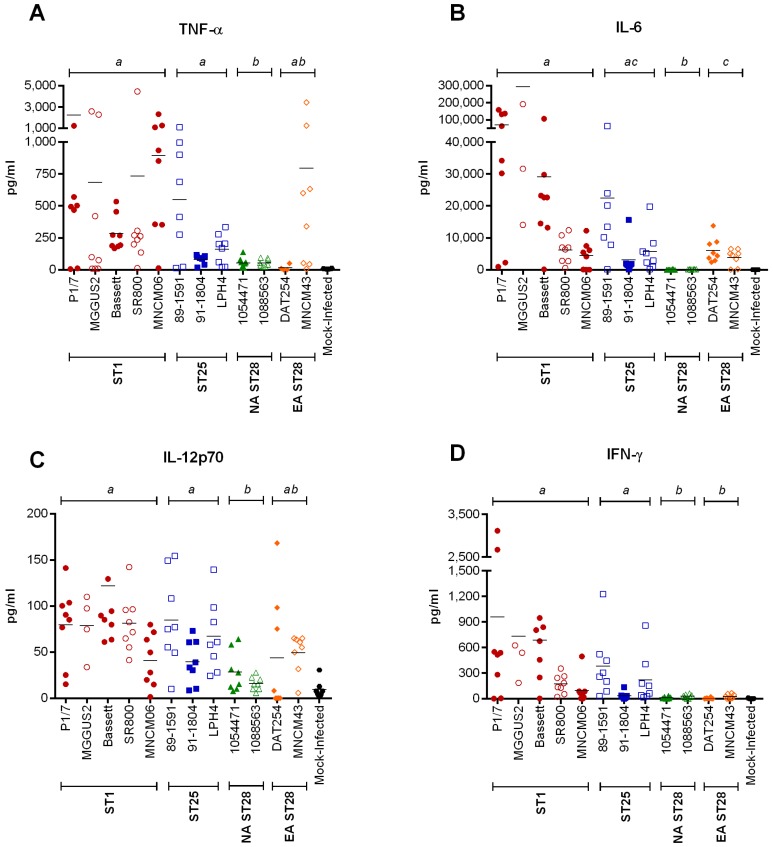
Plasma cytokine production is lowest in North American ST28-infected mice, intermediate in Eurasian ST28-infected mice, and highest in ST1- and ST25-infected mice during systemic infection. Plasma cytokine levels 12 h post-intraperitoneal inoculation of mock- (vehicle) or 5 × 10^7^ CFU of *S. suis*-infected C57BL/6 mice, as determined by Luminex^®^ for TNF-α (**A**); IL-6 (**B**); IL-12p70 (**C**); and IFN-γ (**D**). Data of individual mice are presented as pg/mL with the mean. Significance between groups is indicated by different letters (*p* < 0.05).

**Figure 4:**
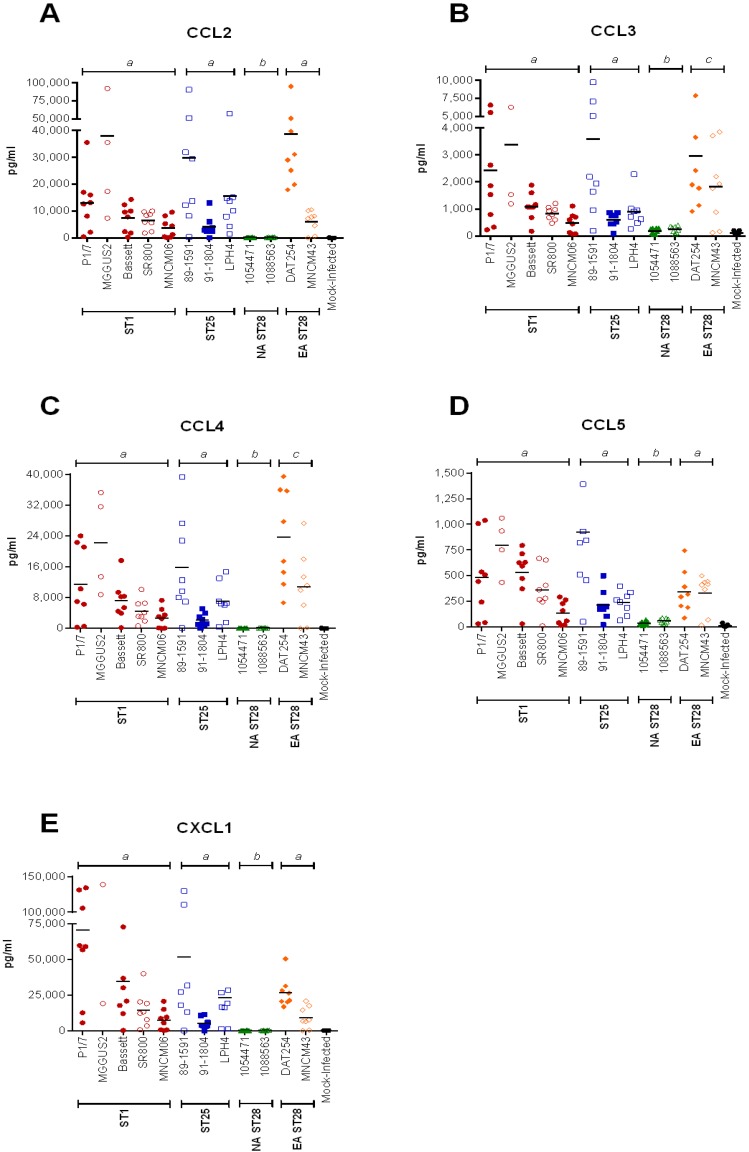
Plasma chemokine production is lowest in North American ST28-infected mice, intermediate in Eurasian ST28-infected mice and highest in ST1- and ST25-infected mice during the systemic infection. Plasma chemokine levels 12 h post-intraperitoneal inoculation of mock- (vehicle) or 5 × 10^7^ CFU of *S. suis*-infected C57BL/6 mice, as determined by Luminex^®^ for CCL2 (**A**); CCL3 (**B**); CCL4 (**C**); CCL5 (**D**), and CXCL1 (**E**). Data of individual mice are presented as pg/mL with the mean. Significance between groups is indicated by different letters (*p* < 0.05).

An exacerbated systemic inflammatory response may be responsible for host death due to *S. suis*-induced septic shock. Consequently, the plasma cytokine and chemokine levels of mice infected with the different *S. suis* serotype 2 strains were determined by Luminex^®^ at 12 h p.i. (Figure 1). This incubation time, chosen based on previous studies with ST1 strains, corresponds to the peak production of these inflammatory mediators in mice following infection with *S. suis* [26,27]. ST1 and ST25 strains induced a significantly higher production of tumor necrosis factor (TNF)-α, interleukin (IL)-6, IL-12p70, and interferon (IFN)-γ than NA ST28 (Figure 3) (*p* < 0.01). Significant differences were also observed between ST1- and EA ST28-infected mice for IL-6 and IFN-γ (*p* < 0.05), between ST25- and EA ST28-infected mice for IFN-γ (*p* < 0.001), and between NA and EA ST28 strains for IL-6 (*p* < 0.001) only. Interestingly, no differences were observed between ST1- and ST25-infected mice, regardless of the cytokine. The plasma concentrations of the chemokines C-C motif ligand (CCL)2, CCL3, CCL4, CCL5, and C-X-C motif ligand (CXCL)1 were also quantified (Figure 4). A significantly higher production in ST1-, ST25-, or EA ST28-infected mice was observed for all chemokines compared to NA ST28-infected mice (*p* < 0.001). Furthermore, significant differences were also observed between ST1- or ST25- and EA ST28-infected mice for CCL3 and CCL4 (*p* < 0.05). As with cytokines, no significant differences were observed between ST1- and ST25-infected mice, regardless of the chemokine. For all of the cytokines and chemokines evaluated, significant differences were observed between ST1-, ST25-, NA, EA ST28-, and mock-infected mice (*p* < 0.05), with the exception of IFN-γ for NA and EA ST28-infected mice. Obtained data indicate that systemic inflammation is implicated in host death during the systemic infection, given that induced mediators could be associated with mortality 72 h p.i. for most strains. However, some exceptions were observed. For example, ST1 strains Bassett and SR800 and ST25 strain LPH4 induced intermediate to high levels of these inflammatory mediators yet caused little mortality. This indicates that although the ST and the geographical origin might present some kind of correlation, exceptions may occur.

### 2.5. Hematogenous-Induced Meningitis: The ST1, ST25 and Eurasian ST28 Strains Induce Similar, Yet Higher Rates of Meningitis in Infected Mice than Do the North American ST28 Strains

Following acute systemic infection, surviving mice are susceptible of developing a CNS disease at later incubation times (Figure 1), characterized by meningitis accompanied or not by encephalitis, as evaluated by the presence of clinical signs (spatial disorientation, hyper-excitement followed by opisthotonos, circular walking with head bent to the side, sudden spinning while in recumbence, and tonicoclonic movements) and histopathology. ST1, ST25, and EA ST28 strains caused significantly more meningitis (20%, 29%, and 36%, respectively) than NA ST28 strains, which induced only 11% of meningitis (*p* < 0.05) (Table 2). As during the systemic infection, and regardless of statistical differences between STs, important variations were also observed amongst strains of the same ST (Table 2).

**Figure 5:**
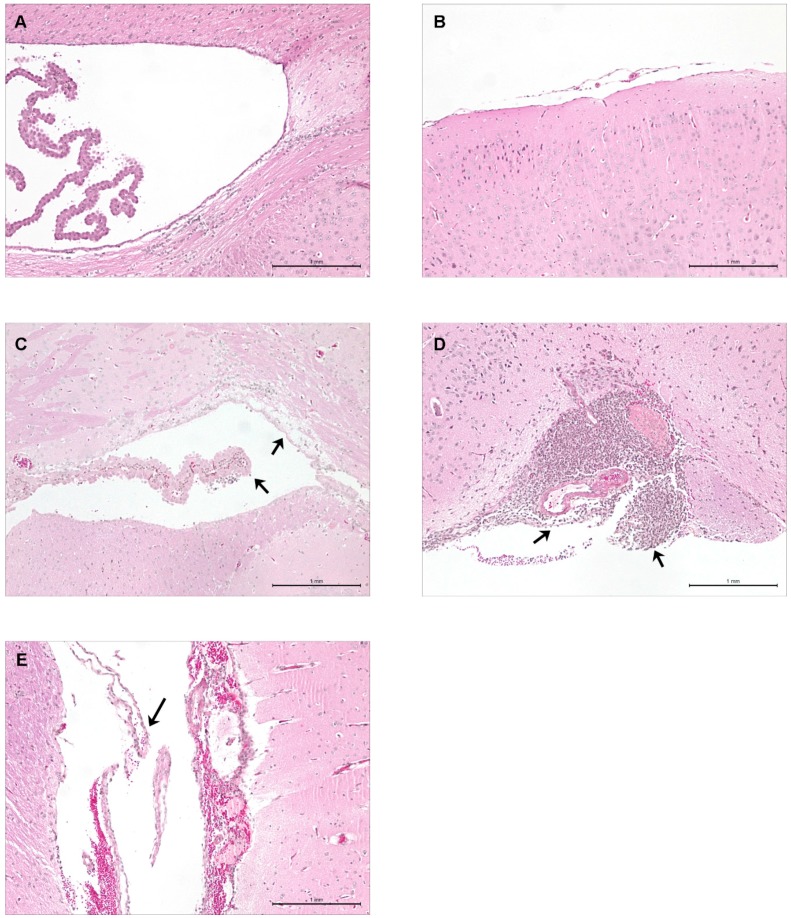
Histopathological studies of the brains of C57BL/6 mice infected by intraperitoneal inoculation during central nervous system infection. Presence or absence of histopathological lesions of meningitis as determined in the brains of mock-infected (vehicle) and infected mice. Micrographs of the meninges or ventricular choroid plexus of mock-infected mice (**A**); NA ST28 strain 1088563- (**B**); EA ST28 strain MNCM43- (**C**); NA ST25 strain 89-1591- (**D**); and EA ST1 strain P1/7- (**E**) infected mice. Black arrowheads indicate lesions typical of *S. suis* meningitis. HPS staining, 100× magnification. NA = North America; EA = Eurasia.

Typical histopathological lesions of mouse meningoencephalitis include multifocal gliosis, meningeal and/or brain suppuration, mononuclear and polymorphonuclear leukocyte infiltration, and brain hemorrhage and necrosis. All ST1, ST25, and EA ST28 strains, as well as certain of the NA ST28 strains tested, induced such lesions. Mice inoculated with Todd-Hewitt broth (THB) (Figure 5A), which served as the vehicle, did not present any clinical signs or histopathological lesions of meningoencephalitis, identical to mice infected with NA ST28 strains that did not induce meningitis (Figure 5B). Meanwhile, mice inoculated with EA ST28 strains (Figure 5C) presented moderate meningeal and brain suppuration, while ST25 strains (Figure 5D) induced severe meningeal and brain suppuration accompanied by brain hemorrhage and necrosis. Brain histopathology of mice inoculated with ST1 strains (Figure 5E) presented similar lesions to those previously reported for an European ST1 strain, 31533 [26]. Multifocal gliosis and leukocyte infiltration were also observed in the brains of mice infected with the ST1, ST25, and EA ST28 strains.

Alongside, blood bacterial burden, evaluated 5 days p.i. (Figure 1), which in this study corresponds to persistent bacteremia, was significantly higher in ST1-, ST25- or EA ST28-infected mice than in NA ST28-infected mice (*p* < 0.05) (data not shown). These persistent titers indicate that ST1, ST25, and EA ST28 strains survived in blood following the systemic infection, while NA ST28 strains generally did not. Using these data, a significant association between the presence of a blood bacterial burden at least 5 days p.i. and the presence of subsequent clinical signs of meningitis in mice infected with *S. suis* serotype 2 was observed (*p* < 0.001).

### 2.6. Brain Bacterial Burden Is Only Present in Infected Mice Following the Development of Meningitis, Regardless of the ST

The presence of bacteria in the brain is considered essential for the subsequent development of meningitis, since it is probably responsible for inducing the local inflammatory brain response. In the absence of clinical signs of meningitis, both mice infected with strains incapable of causing meningitis, i.e. lower virulence NA ST28 strains, and mice infected with strains potentially capable of causing meningitis but that never developed clinical signs of meningitis (data not shown), presented undetectable brain bacterial burdens. Contrarily, in the presence of clinical signs of meningitis (Figure 1), and regardless of the ST, high concentrations of *S. suis* were detected in the brain, ranging between 2 × 10^5^ and 6 × 10^7^ colony forming units (CFU)/g (Table 2).

### 2.7. Lower Virulence North American ST28 Strains Remain Incapable of Causing Meningitis Following Intracisternal Injection into the Cerebrospinal Fluid

**Figure 6:**
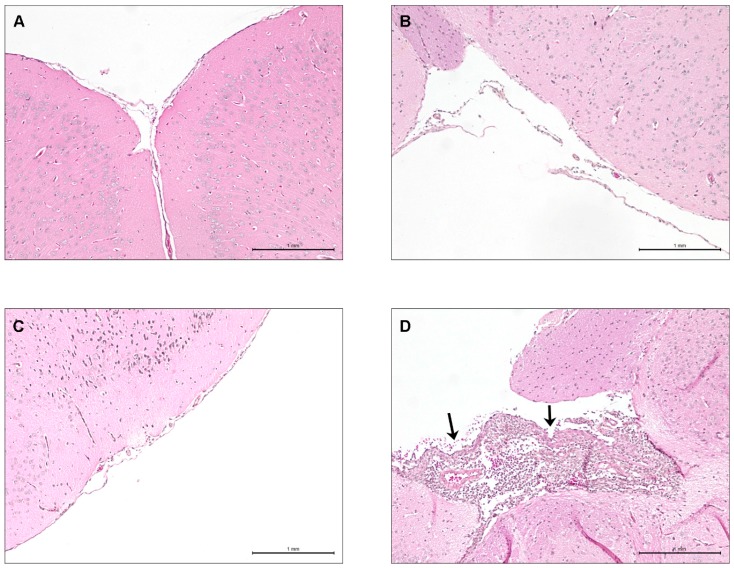
Lower virulence North American ST28 strains do not induce meningitis in C57BL/6 mice following intracisternal inoculation. Presence or absence of histopathological lesions of meningitis in the brains of mock-infected (vehicle) and *S. suis*-infected C57BL/6 mice following intracisternal inoculation. Micrographs of the meninges or ventricular choroid plexus of mock-infected (**A**); NA ST28 strain 1054471- (**B**); NA ST28 strain 1088563- (**C**); and EA ST1 strain P1/7- (**D**) infected mice. Black arrowheads indicate lesions typical of *S. suis* meningitis. HPS staining, 100× magnification. NA = North America; EA = Eurasia.

In the abovementioned experiments, it was observed that certain lower virulence NA ST28 strains (1054471 and 1088563) were incapable of causing meningitis following intraperitoneal inoculation. It remained unclear if this was due to their relative incapacity of surviving in blood or to an intrinsic inability to induce meningitis. To answer this question, the transcutaneal intracisternal inoculation route (Figure 1) was used with a dose of 2 × 10^5^ CFU. Negative (sterile THB or 2 × 10^5^ CFU of heat-killed P1/7) and positive (2 × 10^5^ CFU of P1/7) controls were also included. Previous studies showed that a virulent ST1 strain induced meningoencephalitis at this dose within 24 h p.i. in CD-1 mice [30]. Clinical signs of CNS disease were monitored and brain histopathology was carried out. Mice inoculated with either THB (Figure 6A) or heat-killed bacteria (data not shown) did not present clinical signs of meningitis or histopathological lesions 72 h p.i.; this was also the case for mice inoculated with either of the two lower virulence NA ST28 strains (Figure 6B,C). However, mice infected with the European ST1 strain P1/7 presented both clinical signs and histopathological lesions associated with meningoencephalitis within 24 h p.i. (Figure 6D), including severe meningeal and brain suppuration, multifocal gliosis, leukocyte infiltration, and brain hemorrhage and necrosis.

**Figure 7:**
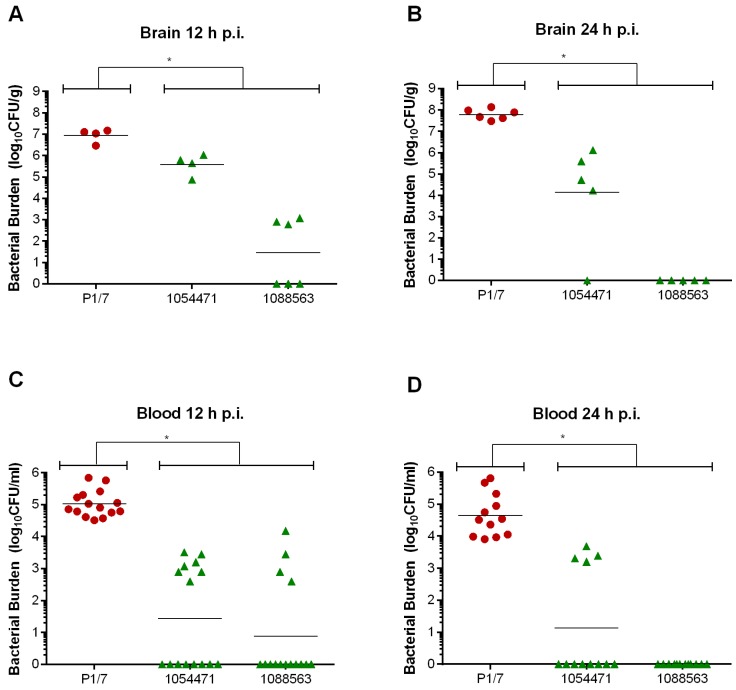
Brain and blood bacterial titers of lower virulence North American ST28 strains are transient, while those of the ST1 strain are persistent, following intracisternal inoculation. C57BL/6 mice were inoculated by intracisternal injection with 2 × 10^5^ CFU and brain and blood bacterial titers evaluated 12 h (**A** & **C**) and 24 h (**B** & **D**) post-infection (p.i.). Data of individual mice are presented as log_10_ CFU/g or CFU/mL with the geometric mean. * Indicates a significant difference between the Eurasian ST1 strain P1/7 and both North American ST28 strains (1054471 and 1088563) (*p* < 0.01).

Since these two NA ST28 strains were incapable of inducing meningitis following intracisternal inoculation, their ability to persist in the brain was evaluated (Figure 1). It was observed that at 12 h p.i. (Figure 7A), brain bacterial burden of mice infected with these NA ST28 strains, which averaged 1 × 10^3^ CFU/g, was significantly lower than that of P1/7-infected mice (*p* < 0.01). At 24 h p.i. (Figure 7B), this difference was even more pronounced: 6 × 10^7^ CFU/g were detected in the brains of P1/7-infected mice compared with nearly undetectable levels (average of 1 × 10^2^ CFU/g) in those of NA ST28-infected mice.

Though blood bacteria burden is generally absent or undetectable upon presentation of clinical signs of meningitis, its presence can result in meningitis-induced complications and worsening of disease [31]. Given the elevated brain bacterial titers detected 12 and 24 h p.i. in P1/7-infected mice, secondary bacteremia following meningitis was evaluated (Figure 1). As of 12 h p.i. (Figure 7C), biologically relevant blood bacterial titers were detected in P1/7-infected mice, which averaged 1 × 10^5^ CFU/mL, and which were significantly higher than those of NA ST28-infected mice (*p* < 0.01). This significant difference was also observed 24 h p.i. (Figure 7D) (*p* < 0.001), at which time blood bacterial titers of P1/7-infected mice remained relatively stable, at 4 × 10^4^ CFU/mL, while those of NA ST28-infected mice were generally undetectable.

### 2.8. S. suis Serotype 2-Induced Pro-Inflammatory Brain Response Only Occurs in the Presence of Meningitis

**Figure 8:**
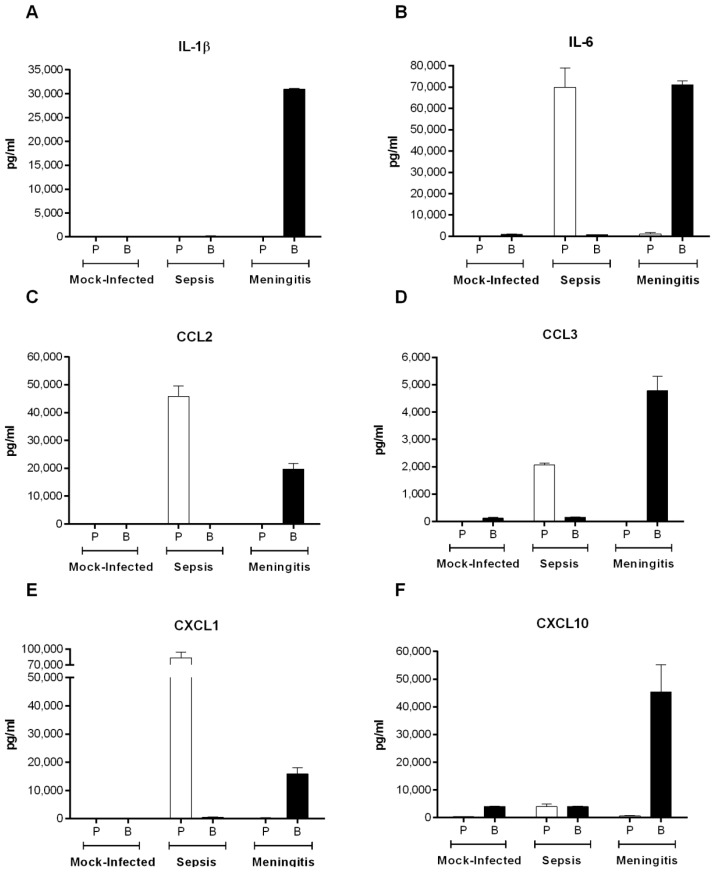
Plasma (P) and brain (B) homogenate cytokine and chemokines levels of mock-infected (vehicle) or C57BL/6 mice inoculated by intraperitoneal injection with *S. suis* serotype 2 European ST1 strain P/7, upon presentation of clinical signs of septic shock or meningitis, by ELISA for IL-1β (**A**); IL-6 (**B**); CCL2 (**C**); CCL3 (**D**); CXCL1 (**E**); and CXCL10 (**F**). Data are presented as mean ± SEM pg/mL.

In situ protein levels of inflammatory mediators have never been quantified during *S. suis* meningitis. Firstly, it was verified that the inflammatory mediators produced in the blood during septic shock and in the brain during meningitis remained compartmentalized and separate as a result of the blood–brain barrier and blood–cerebrospinal fluid (CSF) barrier, and did not seep between systems (Figure 1). Consequently, the levels of IL-1β and IL-6 and chemokines CCL2, CCL3, CXCL1, and CXCL10 were quantified in the plasma and brain homogenates of mice infected with the European ST1 strain P1/7 (Figure 8). Remarkably, complete compartmentalization was observed during septic shock and meningitis in both the plasma and brain for all of mediators tested.

**Figure 9:**
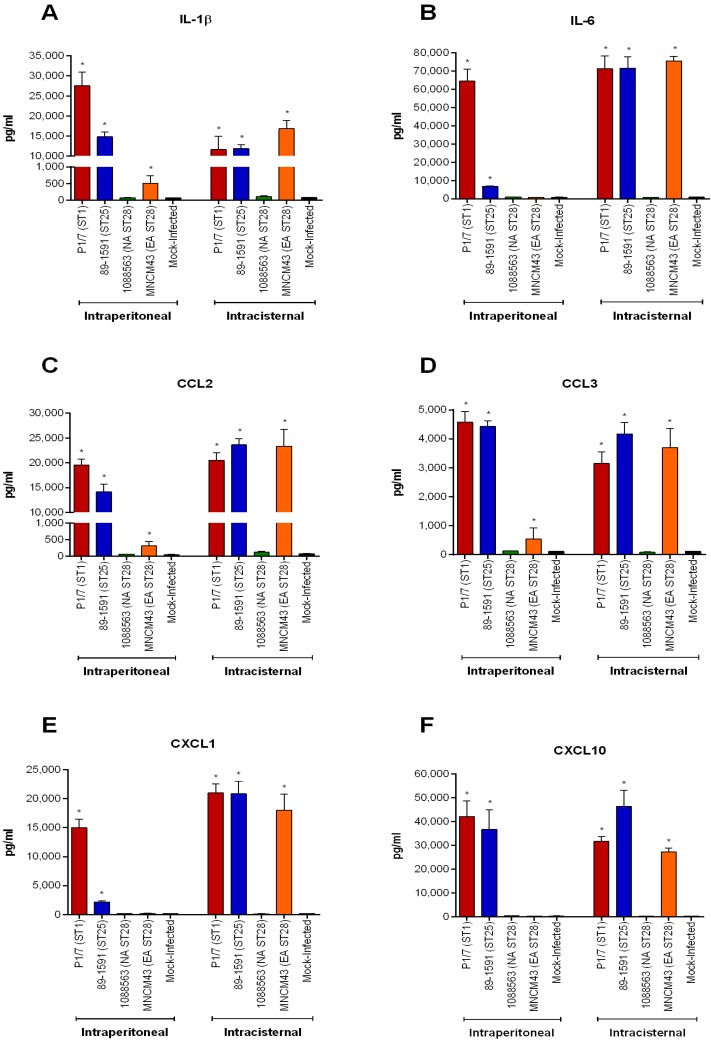
Production of brain cytokines and chemokines following *S. suis* serotype 2 infection only occurs in the presence of meningitis. Brain cytokine and chemokine levels of mock-infected (vehicle) or *S. suis*-infected C57BL/6 mice inoculated by intraperitoneal or intracisternal injection as determined by ELISA. Intraperitoneally injected mice were euthanized upon presentation of clinical signs of meningitis or at the end of the study (14 days post-infection) and intracisternally injected mice 24 h post-infection or at the end of the study (72 h post-infection). Brain levels of IL-1β (**A**); IL-6 (**B**); CCL2 (**C**); CCL3 (**D**); CXCL1 (**E**); and CXCL10 (**F**) following infection with EA ST1 strain P1/7, NA ST25 strain 89-1591, NA ST28 strain 1088563, or EA ST28 strain MNCM43. Data are presented as mean ± SEM pg/mL. * Indicates a significant difference with mock-infected mice (*p* < 0.05). EA = Eurasian; NA = North American.

Subsequently, production of these six mediators was quantified in brain homogenates using representative strains following intraperitoneal (upon presentation of clinical signs of meningitis or at the end of the study for strains incapable of causing meningitis) and intracisternal (24 h p.i. or at the end of the study for strain incapable of causing meningitis) inoculation (Figure 9). Following intraperitoneal inoculation, none of the six pro-inflammatory mediators were detected in the brains of mice infected with the NA ST28 strain 1088563, which did not induce meningitis. Meanwhile, these mediators were highly and significantly induced in mice with meningitis and infected with the ST1 strain P1/7 and ST25 strain 89-1591, when compared with mock-infected mice (*p* < 0.001). Intermediately, the EA ST28 strain MNCM43, which caused meningitis, induced a significant production of IL-1β, CCL2, and CCL3 only (*p* < 0.05). These results were compared with those from mice infected by intracisternal inoculation. Once again, none of the six pro-inflammatory mediators tested could be detected in the brains of mice infected with the NA ST28 strain 1088563, which did not induce meningitis. Similar results were also obtained for the NA ST28 strain 1054471 (data not shown). However, these mediators were highly and significantly induced in the brains of mice infected with the strains P1/7 (ST1), 89-1591 (ST25), and MNCM43 (EA ST28) when compared with mock-infected mice (*p* < 0.05) (Figure 9). Interestingly, and differently from what was observed after intraperitoneal inoculation, similar levels of inflammatory mediators were detected in the brains of mice infected with these three strains. In fact, the Eurasian ST28 strain MNCM43 induced significantly higher levels of cytokines and chemokines following intracisternal inoculation when compared to intraperitoneal inoculation, regardless of presenting similar clinical signs and histopathological lesions of meningoencephalitis than the ST1 and ST25 strains P1/7 and 89-1591, respectively.

## 3. Discussion

*S. suis* serotype 2, an important porcine pathogen and emerging zoonotic agent, is the most widespread of the described *S. suis* serotypes worldwide [2]. Recent studies have associated archetypal strains of different *S. suis* serotype 2 STs with virulence [16,19]. However, the virulence potential of strains belonging to the same ST but from different geographical and host origins had never been investigated. In the present study, virulence of different strains was evaluated in an inbred mouse model of infection in order to reduce variability due to host heterogeneity. In addition, the predictive virulence potential of strains was also tested using in vitro assays.

Since animal models of infections are not the most straightforward approach to predict *S. suis* virulence, both logistically and ethically, different well-described in vitro assays have been developed. For example, the capacity to resist phagocytosis by immune cells, which has been largely described as being an essential step in the pathogenesis of *S. suis* serotype 2, is often associated with virulence [1]. In the present study, a clear relationship between resistance to phagocytosis by murine macrophages and virulence in the mouse model could not be established. Conditions used for phagocytosis assays in previously published studies are highly variable. Different cell types (dendritic cells, macrophages, monocytes, and neutrophils), hosts (human, mouse, and pig) and the absence or presence of normal or heat-inactivated serum have been used [32,33,34]. It may be hypothesized that in vitro conditions used for phagocytosis assays can influence the outcome of the test. For example, results obtained in the current study using the ST1 strain P1/7 in the presence of 10% serum showed clearly higher rates of internalization than those previously reported with the same cells in the absence of serum [33,35]. However, whether complement or other host serum factors are responsible for this effect is not clear. In fact, the role of complement during the systemic infection caused by *S. suis* is still controversial. While an early report suggested a limited role of complement in phagocytosis and killing of well-encapsulated *S. suis* [36], another study showed that the complement system contributes to limit *S. suis* invasiveness at early stages of the infection [37]. Nevertheless, the role of complement and other conditions in *S. suis* phagocytosis tests remain to be confirmed. Finally, phagocytosis results are usually evaluated at one specific time point. The possibility that levels of phagocytosis are similar among the strains but that certain strains are rapidly killed intracellularly cannot be ruled out.

On the other hand, a blood bactericidal assay, which more closely resembles the systemic conditions in which *S. suis* encounters different phagocytic cells and blood components [1], provided results different from those of the phagocytosis test and more closely correlated with in vivo virulence. However, correlation was only partial. For example, certain strains (Bassett, SR800, and LPH4) were highly resistant to killing by mouse blood but presented, in general, low virulence in vivo. It is possible that, as in the case with phagocytosis, the increasingly popular killing test [38,39,40] also needs to be optimized and compared with in vivo virulence to be validated. Confirmation of virulence of a given strain in an animal model of infection remains a requirement [16,26,27,28].

Based on a study with archetypal strains, it was previously hypothesized that ST1 strains were more virulent than NA ST25, the latter being more virulent than NA ST28 strains. In addition, it was also hypothesized that EA ST28 strains may be virulent, differently from their NA counterparts. Results obtained in this study partially support this hypothesis, which might explain, to a certain extent, the greater number of serotype 2 cases recorded in Europe and in some Asian countries than in North America [2,41]. This is especially true with ST28 strains: NA strains caused little or no mortality during the systemic infection while EA strains were virulent. Results with ST28 strains support a certain level of genetic heterogeneity of these strains as determined by whole-genome sequencing, whereby NA and EA ST28 strains clustered separately [23]. However, a critical analysis shows that, regardless of statistical differences, important variations in mortality were observed within STs. This indicates that, for strains other than NA ST28, it would be dangerous to assume that a given strain is virulent or non-virulent based only on its background. It would be important to confirm such observations using a higher number of strains. Finally, virulence of tested strains isolated from either porcine or human clinical cases of infection was similar, which supports previous genetic analyses [8].

It has been proposed that mortality during the systemic infection caused by *S. suis* may be the result of a high and uncontrolled bacteremia combined with an exacerbated pro-inflammatory response [26,27]. For the first time, this study demonstrates that mortality is positively associated with both bacteremia and plasma pro-inflammatory mediators during the acute systemic infection for strains other than archetypal ST1 [26] and ST25 strains [16]. Consequently, inflammatory mediators are a reflection of the host response to bacteremia. Following the systemic infection, surviving individuals are susceptible of developing a CNS infection characterized by meningitis accompanied or not by encephalitis [26]. Results also showed for the first time that meningitis can be induced by a strain belonging to a ST other than the ST1 in the mouse model of infection.

In general, virulent ST1, ST25, and EA ST28 strains persisted in circulation, whereas NA ST28 strains were mostly undetectable by the end of the systemic infection. This suggests that the latter strains are less resistant to killing, confirming results obtained using the whole blood assay in vitro. Similarly, ST1, ST25, and EA ST28 strains induced higher rates of meningitis, while most NA ST28 strains did not induce important disease. It has been demonstrated for human meningitis-inducing bacterial pathogens that a high acute blood bacterial burden is a predisposing factor for the development of meningitis [42,43]. However, this association had not been determined with regards to persistent bacteremia, and never for *S. suis*. Differently from NA ST28 strains, persistent bacteremia was observed with ST1, ST25, and EA ST28 strains, all of which induced important, yet similar rates of meningitis. This indicates that persistent *S. suis* serotype 2 bacteremia is a prerequisite for the subsequent development of meningitis. Thus, if bacteremia can be rapidly cleared, the risk of developing subsequent meningitis, which is often more difficult to treat [41], is greatly reduced.

Alongside, excessive inflammation was also identified as a crucial step in systemic virulence [16,26]. While ST1 and ST25 strains induced the highest levels of pro-inflammatory mediators, EA ST28 strains induced mostly chemokines, which may suggest the utilization of different mechanisms to cause inflammation and host death. In accordance, a rapid clearance of NA ST28 bacteria from the bloodstream may explain the lack of pro-inflammatory mediators induced by such strains.

Brain bacterial burden is, for most meningitis-causing bacterial pathogens, considered essential and responsible for inducing the local inflammatory response [42,44]. Indeed, our results demonstrated that the absence or presence of meningitis is associated with the absence or presence of a minimum threshold of *S. suis* brain burden, respectively. Previous results with *Streptococcus pneumoniae* showed that elevated brain bacterial burden responsible for meningitis can, from the CSF, enter the bloodstream: this event may lead to secondary bacteremia, resulting in systemic host inflammation and decreased host response during treatment [31,45]. Indeed, results obtained in the current study revealed that *S. suis* meningitis can result in secondary bacteremia, which could worsen the outcome of the infection. On the other hand, the NA ST28 strains tested that did not induce meningitis via the hematogenous route of infection were also unable to induce meningitis following inoculation into the CSF. This suggests that lower virulence strains cannot persist in the CNS even if they manage to bypass clearance by blood phagocytic cells. Similarly, these strains also seem susceptible to elimination by resident CNS cells [46,47]. Although less virulent in mouse and pig models of infection [4,48], porcine cases of infection caused by NA *S. suis* serotype 2 strains occur in the field, suggesting the implication of environmental, management, and/or co-infection factors [25]. These stressful conditions often result in increased susceptibility to infections by secondary pathogens, such as *S. suis*, including by less virulent strains [25].

The inflammation responsible for the development of *S. suis*-induced meningitis has been barely studied. It was previously observed that mRNA levels of a few pro-inflammatory mediators are upregulated in the brains of mice during meningitis [26], but protein levels have never been quantified. In the present study, a pro-inflammatory brain response was only observed in the presence of meningitis, regardless of the ST, which suggests a probable implication of inflammatory cells in *S. suis*-induced meningitis. Accordingly, there was an absence of brain mediator production following infection with lower virulence NA ST28 strains, even when injected directly in the CNS, probably as a consequence of their reduced resistance to the local defense systems. Interestingly, production of IL-1β was clearly observed for the first time during the *S. suis* meningitis; levels of this cytokine are barely quantifiable during the systemic infection [26]. This cytokine may play an important role during the *S. suis* meningitis, as has been reported for *S. pneumoniae*, where levels of IL-1β determine the outcome and severity of the disease [49]. Albeit the source of *S. suis*-induced IL-1β remains unknown, in vitro studies have previously demonstrated that microglia and astrocytes are not responsible for this production [50,51]. Infiltrating cells may be the source of this cytokine since monocytes have been shown to produce IL-1β when activated with *S. suis* [52]*.* Moreover, the production of chemokines (such as CCL2, CCL3, and CXCL1), possibly produced by resident CNS cells [50,51], may be responsible for the infiltration of blood leukocytes into the brain. Of note, similar to what has been observed in the bloodstream, the EA ST28 strain induced higher levels of chemokines than cytokines in the CNS. Finally, a massive production of IL-6 was also detected. When produced in the brain during *S. pneumoniae* meningitis, IL-6 is responsible for increased intracranial pressure [53]. Production of pro-inflammatory cytokines, which are known to induce neuronal death [54], are possibly responsible for the histopathological lesions observed during the *S. suis* meningitis, including brain hemorrhaging and necrosis.

Interestingly, the present results showed that systemic mediators in blood during septic shock and brain mediators in the CNS during meningitis remained completely separated and compartmentalized during both types of *S. suis* infections. Though not yet demonstrated, an important increase of brain barrier permeability possibly occurs following *S. suis* infection given the massive infiltration of leukocytes during meningitis [26]. Consequently, this observation was completely unexpected. To our knowledge, this is the first time that such a compartmentalization has ever been demonstrated. A retrospective study of meningococcal infection determined that CSF mediators remained separated from plasma during meningitis, but not septic shock [55]. Furthermore, CSF but not brain mediators were evaluated. As such, it may be hypothesized that pro-inflammatory mediators are highly concentrated in the CNS during meningitis and diluted in the bloodstream, which might explain the low systemic IL-1β levels detected during *S. suis* infection.

## 4. Materials and Methods

### 4.1. Bacterial Strains and Growth Conditions

The different well-encapsulated *S. suis* serotype 2 strains used in this study are listed in Table 3. Serotyping was performed with the coagglutination test using serotype 1 and 2 reference antisera, as previously described, to confirm that the strains used were serotype 2 and not serotype 1/2 [56]. Strains giving a strong positive reaction within 30 s to serotype 2 only were used, as a clear indication of presence of serotype-specific capsular polysaccharide. In addition, and for comparison purposes, an isogenic non-encapsulated mutant derived from strain P1/7, Δ*cpsF* [57], was also included during in vitro experiments. Bacteria were grown overnight on Columbia agar supplemented with 5% sheep blood (Oxoid, Nepean, ON, Canada) at 37 °C with 5% CO_2_. Five milliliters of THB (Becton Dickinson, Mississauga, ON, Canada) were inoculated with isolated colonies and incubated for 8 h at 37 °C with 5% CO_2_. Working cultures were prepared by inoculating 30 mL of THB with 10 µL of a 10^−3^ dilution of the 8 h cultures and incubating for 16 h at 37 °C with 5% CO_2_. Bacteria were washed twice with pH 7.3 phosphate-buffered saline (PBS) and resuspended in cell culture medium (phagocytosis and bactericidal assays) or THB (experimental mouse infections), appropriately diluted, and plated on Todd Hewitt broth agar (THA) to accurately determine bacterial concentrations using an Autoplate 4000 Spiral Plater (Spiral Biotech, Norwood, MA, USA).

### 4.2. S. suis MLST

Genomic DNA of the *S. suis* serotype 2 strain SR800 was prepared and MLST performed as previously described by sequencing the *cpn60*, *dpr*, *recA*, *aroA*, *thrA*, *gki*, and *mutS* housekeeping genes [19]. This strain was determined to be ST1 using the *S. suis* Multilocus Sequence Typing Database [62]. The STs of the other strains have been previously published [19,63,64].

### 4.3. S. suis Serotype 2 Phagocytosis Assay

Phagocytosis assays were performed as previously described [65] with some modifications. J774A.1 murine macrophages (ATCC TIB-67; Rockville, MD, USA) were maintained in Dulbecco’s Modified Eagle’s Medium (Gibco, Burlington, ON, Canada) supplemented with 10% heat-inactivated fetal bovine serum (Gibco) and 0.25% 10,000 U penicillin/streptomycin (Gibco), and cells grown at 37 °C with 5% CO_2_. Confluent cell cultures were scraped, washed twice with PBS, suspended in antibiotic-free medium at 1 × 10^5^ cells/mL, and incubated for 3 h at 37 °C with 5% CO_2_ to allow cell adhesion. Cells were infected by adding 1 × 10^7^ CFU/mL of bacterial suspension in complete culture medium containing 10% of fresh mouse serum, without antibiotics (multiplicity of infection; MOI = 100). The infected cells were incubated for 1 h at 37 °C with 5% CO_2_ to allow optimal phagocytosis, determined during preliminary studies (data not shown). After incubation, cell monolayers were washed twice with PBS and incubated with medium containing 5 µg/mL penicillin G (Sigma-Aldrich, Oakville, ON, Canada) and 100 µg/mL of gentamicin (Gibco) for 1 h to kill extracellular bacteria. Supernatant controls were taken during every test to confirm the activity of the antibiotics. After antibiotic treatment, cell monolayers were washed three times with PBS, lysed with water and vigorous pipetting, and viable intracellular bacteria determined by plating appropriate dilutions as described above. Each test was repeated three times in independent experiments and the number of CFU/mL was determined. Fresh mouse serum was obtained from six- to ten-week-old female C57BL/6 mice (Charles River Laboratories, Wilmington, MA, USA).

### 4.4. S. suis Serotype 2 Bactericidal Assay

Blood was collected from six- to ten-week-old female C57BL/6 mice (Charles River Laboratories) and mixed with sodium heparin (Sigma-Aldrich). Blood was adjusted with RPMI-1640 (Gibco) to obtain 6 × 10^6^ leukocytes/mL. Leukocytes (1 × 10^6^ cells) were transferred to a microtube containing 1 × 10^6^ CFU of the different *S. suis* strains (MOI = 1) and incubated for 4 h, mixing every 20 min. Assay conditions were chosen based on preliminary assays of the kinetics of *S. suis* killing by murine blood (data not shown). After incubation, cells were lysed with water and vigorous pipetting, and appropriate dilutions plated on THA to determine viable bacterial counts as described above. Resistance to bacterial killing by blood leukocytes was compared to incubation of the different bacterial strains in plasma only (similarly diluted in RPMI-1640). Each test was repeated at least three times in independent experiments and the percentage of bacteria killed determined using the following formula: 1 − (bacteria in blood /bacteria in plasma) × 100%.

### 4.5. S. suis Serotype 2 Experimental Mouse Infections

Throughout this study, six-week-old C57BL/6 mice (Charles River Laboratories) were used. A total of 650 mice were required to complete this study. To further reduce host variability, only female mice were used. Mice were acclimatized to standard laboratory conditions with unlimited access to water and rodent chow [27]. These studies were carried out in strict accordance with the recommendations of and approved by the University of Montreal Animal Welfare Committee (protocol number rech-1570) guidelines and policies, including euthanasia to minimize animal suffering, which was applied throughout this study when animals were seriously affected since mortality was not an endpoint measurement. Antibiotic treatment, previously reported as not being required in the *S. suis* mouse model of infection [26,27], was not administered regardless of the route of inoculation and phase of infection. For systemic virulence studies, 5 × 10^7^ CFU of the different *S. suis* serotype 2 strains, or the vehicle solution (sterile THB), were administered by intraperitoneal inoculation to groups of 15 mice. Mice were monitored at least three times daily until 72 h p.i. Mortality recorded at this time point was considered to be due to systemic infection. For the hematogenous meningitis model, animals were infected with 5 × 10^7^ CFU or with 2 × 10^7^ CFU for strains presenting higher virulence; all animals were monitored at least twice daily until the end of the study (14 days p.i.) for clinical signs and mortality. For the transcutaneal intracisternal model of meningitis, groups of 10 mice were anesthetized with inhaled isofluorane (Pharmaceutical Partners of Canada, Richmond Hill, ON, Canada) and 20 μL of the different *S. suis* serotype 2 strains (final concentration of 2 × 10^5^ CFU) were injected as previously described [30]. Animals were allowed to wake up, monitored every 6 h, and euthanized upon presentation of clinical signs of meningitis or at the end of the study (72 h p.i.). Controls were injected with 20 μL of the vehicle solution (sterile THB). A timeline presenting the intraperitoneal (Figure 1A) and transcutaneal intracisternal (Figure 1B) models of inoculation used in this study is included.

### 4.6. Measurement of Mouse Blood Bacterial Burden

Blood bacterial titers were assessed in surviving mice 24 h, 36 h, 48 h, and five days p.i. following intraperitoneal inoculation, and 12 h and 24 h p.i. following intracisternal inoculation, by collecting 5 µL of blood from the tail vein. Proper dilutions were plated as described above.

### 4.7. Measurement of Mouse Plasma (Systemic) Cytokine and Chemokine Levels

A total of eight mice per strain were infected as described above and blood was collected from surviving mice euthanized 12 h p.i. by intracardiac puncture and stabilized with EDTA (Sigma-Aldrich) as previously described [16]. Plasma supernatants were collected following centrifugation at 10,000× *g* for 10 min, 4 °C, and stored at −80 °C. Plasmatic concentrations of TNF-α, IL-6, IL-12p70, IFN-γ, CCL2 (MCP-1), CCL3 (MIP-1α), CCL4 (MIP-1β), CCL5 (RANTES), and CXCL1 (KC) were determined using a custom-made cytokine Milliplex panel (Millipore) according to the manufacturer’s instructions. Acquisition was performed on the MAGPIX platform (Luminex^®^) and data analyzed using the MILLIPLEX Analyst 5.1 software (Upstate Group/Millipore, Etobicoke, ON, Canada).

### 4.8. Mouse Brain Histopathological Studies

Upon presentation of clinical signs of meningitis or at the end of the study, mice were euthanized and brains recovered and fixed in 10% buffered formalin. After paraffin embedding, 4 µm-thick sections of the brain were stained with hematoxylin phloxine saffron (HPS) according to standard protocol and examined under light microscopy.

### 4.9. Measurement of Mouse Brain Bacterial Burden

Following intraperitoneal inoculation and upon presentation of clinical signs of meningitis or at the end of the study, mice were euthanized and brains aseptically recovered. This was also completed 12 h and 24 h p.i. or at the end of the study following intracisternal inoculation in groups of four to six mice. Brains were homogenized in PBS and bacterial burdens determined by plating appropriate dilutions on THA as described above.

### 4.10. Evaluation of Systemic and Brain Compartmentalization and Measurement of Mouse Brain Cytokine and Chemokine Levels

Systemic and brain compartmentalization was evaluated following intraperitoneal inoculation of strain P1/7. Upon presentation of clinical signs of either septic shock or meningitis, mice were euthanized and plasma recovered as described above. Brains were recovered and frozen in liquid nitrogen. Extraction buffer, prepared using cOmplete Mini, EDTA-free, protease inhibitor cocktail tablets (Roche Diagnostics GmbH, Mannheim, Germany) according to the manufacturers’ instructions and by adding 0.4% (w/v) CHAPS (Sigma-Aldrich), was added to the brains, which were homogenized using a POLYTRON PT 1200E system bundle (Kinematica, Lucerne, Switzerland). Brain homogenate supernatants were collected following centrifugation at 10,000× *g* for 10 min, 4 °C, and stored at −80 °C. Levels of IL-1β, IL-6, CCL2 (MCP-1), CCL3 (MIP-1α), CXCL1 (KC), and CXCL10 (IP-10) were determined by a sandwich enzyme-linked immunosorbent assay (ELISA), using pair-matched antibodies (R&D Systems, Minneapolis, MN), as previously described [57] in both plasma and brain homogenates. Once compartmentalization was determined, brain levels of these same mediators were evaluated for the other strains following intraperitoneal (upon presentation of clinical signs of meningitis or at the end of the study) and intracisternal (24 h p.i.) inoculation (*n* = 3 or 4 mice).

### 4.11. Statistical Analyses

The normality of data was verified using the Shapiro–Wilk test. Accordingly, parametric (unpaired *t*-test) or non-parametric tests (Mann–Whitney rank sum test and one-way ANOVA on ranks), where appropriate, were performed to find statistical differences between groups. The log-rank (Mantel–Cox) and chi-square tests were used to compare survival and meningitis rates of studied groups, and to determine associations between blood bacterial burden and presence or absence of clinical signs of meningitis, respectively (*p* < 0.05 was considered statistically significant).

## 5. Conclusions

Overall, this study demonstrates that although a certain tendency is observed, *S. suis* serotype 2 virulence is difficult to predict based on ST and geographical origin alone, since some strains belonging to the same ST presented important differences in virulence, which did not always correlate with a given continent. The only exception appears to be NA ST28 strains, which induce low and transient bacteremia and low production of inflammatory mediators (in the bloodstream and in the CNS) and were generally less virulent in both systemic and CNS infection models. We have also shown that serotype 2 strains belonging to other STs than the ST1 are able to induce meningitis in the mouse model of infection. In addition, persistent and high levels of bacteremia accompanied by an elevated CNS inflammatory reaction are needed for a strain to cause meningitis. Although widely used, in vitro tests such as the phagocytosis assay must be standardized in order for these to be predictive tests that may evaluate the virulence capacities of a given strain. The blood bacterial killing assay better predicts bacteremia and virulence, though as with the phagocytosis assay, standardization is required given that correlation with virulence was only partial. The use of strains other than archetypal strains has increased our knowledge and understanding of the *S. suis* serotype 2 population dynamics. Results obtained in the present study should, ideally, be confirmed using a greater number of strains, as well as by testing representative strains in appropriate experimental infection models in susceptible pigs.

## Figures and Tables

**Table 1:** Resistance to phagocytosis and bacterial killing, as well as the capacity to cause mouse mortality during the systemic infection following intraperitoneal inoculation, by different *S. suis* serotype 2 strains.

Strain	ST	Origin	Phagocytosis (CFU/mL ± SEM) ^1^	Bacterial Killing (% ± SEM) ^2^	Mouse Mortality 72 h p.i. (%) ^3^
P1/7	1	EA	2 ± 0.7 ×10^4^	0 ± 0	67
Δ*cpsF*	-	-	2 ± 0.5 × 10^5^ ***^a^***	64 ± 5 ***^b^***	-
MGGUS2	1	NA	2 ± 0.9 × 10^4^	0 ± 0	80
Bassett	1	NA	2 ± 1 × 10^4^	0 ± 0	7
SR800	1	SA	3 ± 0.9 × 10^4^	0 ± 0	0
MNCM06	1	EA	2 ± 0.9 × 10^4^	0 ± 0	20
89-1591	25	NA	8 ± 5 × 10^4^	20 ± 5	47
91-1804	25	NA	1 ± 0.4 × 10^4^	34 ± 5	0
LPH4	25	EA	6 ± 2 × 10^4^	2 ± 2	7
1054471	28	NA	3 ± 0.5 × 10^3^	33 ± 6	0
1084708	28	NA	8 ± 2 × 10^3^	27 ± 3	0
1088563	28	NA	6 ± 0.3 × 10^3^	30 ± 3	0
1097205	28	NA	8 ± 1 × 10^3^	27 ± 2	10
DAT254	28	EA	6 ± 0.9 × 10^3^	3 ± 2	73
MNCM43	28	EA	5 ± 3 × 10^3^	21 ± 3	33

^1^ Phagocytosis of the different strains by murine macrophages after 1 h of incubation with 10% murine serum and a MOI = 100; *^a^* Significant difference between P1/7 and Δ*cpsF* (*p* < 0.001); ^2^ Bacterial killing of the different strains by murine whole blood after 4 h of incubation with a MOI = 1; *^b^* Significant difference between P1/7 and Δ*cpsF* (*p* < 0.001); ^3^ Mortality until 72 h post-infection (p.i.), considered as due to systemic infection; EA = Eurasia; NA = North America; SA = South America.

**Table 2:** Capacity to induce meningitis and brain bacterial burden after the CNS infection following intraperitoneal inoculation by different *S. suis* serotype 2 strains.

Strain	ST	Origin	Meningitis (%) ^1^	Brain Burden (CFU/g) ^2^
P1/7	1	EA	31	6 × 10^7^
MGGUS2	1	NA	20	2 × 10^7^
Bassett	1	NA	14	ND
SR800	1	SA	27	ND
MNCM06	1	EA	8	ND
89-1591	25	NA	20	2 × 10^5^
91-1804	25	NA	27	ND
LPH4	25	EA	36	2 × 10^6^
1054471	28	NA	0	0
1084708	28	NA	20	2 × 10^5^
1088563	28	NA	0	0
1097205	28	NA	11	9 × 10^5^
DAT254	28	EA	33	2 × 10^6^
MNCM43	28	EA	30	3 × 10^6^

^1^ Mice inoculated with 5 × 10^7^ CFU for all strains except for P1/7, MGGUS2 and 89-1591, which were inoculated with 2 × 10^7^ CFU to ensure survival of a sufficient number of mice following the systemic infection; ^2^ Brain burden was determined upon presentation of clinical signs of meningitis or at the end of the study (14 days post-infection); EA = Eurasia; NA = North American; SA = South America; ND = Not determined.

**Table 3:** *S. suis* serotype 2 strains used in this study.

Strain	Sequence Type (ST)	Country	Clinical Feature	Reference
P1/7	1	United Kingdom	Pig, meningitis	[58]
MGGUS2	1	United States	Pig, meningitis	[19]
Bassett	1	United States	Human, meningitis	[20]
SR800	1	Argentina	Human, meningitis	[59]
MNCM06	1	Thailand	Human, meningitis	[21]
89-1591	25	Canada	Pig, sepsis/meningitis	[60]
91-1804	25	Canada	Human, endocarditis	[61]
LPH4	25	Thailand	Human, sepsis	[21]
1054471	28	Canada	Pig, meningitis	[19]
1084708	28	Canada	Pig, sepsis	[19]
1088563	28	Canada	Pig, meningitis	[19]
1097205	28	Canada	Pig, meningitis	[19]
DAT254	28	Japan	Pig, meningitis	[6]
MNCM43	28	Thailand	Human, endocarditis	[21]

## Abbreviations

The following abbreviations are used in this manuscript:

CCL	C-C motif ligand
CFU	Colony forming unit
CNS	Central nervous system
CSF	Cerebrospinal fluid
CXCL	C-X-C motif ligand
EA	Eurasia
ELISA	Enzyme-linked immunosorbent assay
HPS	Hematoxylin phloxine saffron
IL	Interleukin
IFN	Interferon
MLST	Multilocus sequence typing
MOI	Multiplicity of infection
NA	North America
p.i.	Post-infection
PBS	Phosphate-buffered saline
SEM	Standard error of the mean
ST	Sequence type
THA	Todd-Hewitt broth agar
THB	Todd-Hewitt broth
TNF	Tumor necrosis factor
